# AAV-mediated expression of HLA-G1/5 reduces severity of experimental autoimmune uveitis

**DOI:** 10.1038/s41598-019-56462-3

**Published:** 2019-12-27

**Authors:** Elizabeth Crabtree, Liujiang Song, Telmo Llanga, Jacquelyn J. Bower, Megan Cullen, Jacklyn H. Salmon, Matthew L. Hirsch, Brian C. Gilger

**Affiliations:** 1 College of Veterinary Medicine, North Carolina State University, Raleigh, NC USA; 2 Department of Pediatrics, Hunan Normal University Medical College, Changsha, Hunan China; 3 Ophthalmology, University of North Carolina, Chapel Hill, NC USA; 4 Gene Therapy Center, University of North Carolina, Chapel Hill, NC USA; 5 Lineberger Comprehensive Cancer Center, University of North Carolina, Chapel Hill, NC USA

**Keywords:** Diseases, Eye diseases, Uveal diseases

## Abstract

Non-infectious uveitis (NIU) is an intractable, recurrent, and painful disease that is a common cause of vision loss. Available treatments of NIU, such as the use of topical corticosteroids, are non-specific and have serious side effects which limits them to short-term use; however, NIU requires long-term treatment to prevent vision loss. Therefore, a single dose therapeutic that mediates long-term immunosuppression with minimal side effects is desirable. In order to develop an effective long-term therapy for NIU, an adeno-associated virus (AAV) gene therapy approach was used to exploit a natural immune tolerance mechanism induced by the human leukocyte antigen G (HLA-G). To mimic the prevention of NIU, naïve Lewis rats received a single intravitreal injection of AAV particles harboring codon-optimized cDNAs encoding HLA-G1 and HLA-G5 isoforms one week prior to the induction of experimental autoimmune uveitis (EAU). AAV-mediated expression of the HLA-G-1 and -5 transgenes in the targeted ocular tissues following a single intravitreal injection of AAV-HLA-G1/5 significantly decreased clinical and histopathological inflammation scores compared to untreated EAU eyes (p < 0.04). Thus, localized ocular gene delivery of AAV-HLA-G1/5 may reduce the off-target risks and establish a long-term immunosuppressive effect that would serve as an effective and novel therapeutic strategy for NIU, with the potential for applications to additional ocular immune-mediated diseases.

## Introduction

Uveitis is an inflammation of the iris, ciliary body, and choroid associated with both infectious and non-infectious causes^1–4^. Non-infectious uveitis (NIU) is a painful disease that causes blindness in approximately 300,000 people annually in the United States and an estimated 25% of all equines^5,6^. Normally, the blood ocular barrier limits the immune response to intraocular antigens. Any disruption of this barrier, either from systemic disease, trauma or inflammation, can cause this barrier to become leaky and allow blood products and inflammatory cells to enter the eye^1,7,8^. The disrupted barrier also enables various host immune responses to react to ocular self-antigens that are not normally recognized by the immune system. Clinical NIU is a lifelong disease process that often develops spontaneous recurrent bouts of inflammation, likely from T cells recognizing additional autoantigens in the ocular tissue^3,9^. Conventional treatment of NIU is non- specific, including frequent use of topical and/or systemic corticosteroids and other immunosuppressive agents; none of which are effective in preventing uveitis relapses. Long-term systemic corticosteroids and other immunomodulating drugs can be used to prevent uveitis flares, but are often inadequate, and are not preferred due to adverse effects^10^, such as glaucoma, cataract, hypertension, infection, and diabetes, all of which may contribute to development of blindness and systemic illness, even death^1,2,11^.

Experimental autoimmune uveitis (EAU) animal models have been useful in the study of NIU. EAU is mediated predominantly by CD4+ T-lymphocytes, and proinflammatory cytokines, which is very similar to the inflammatory profile in clinical NIU in humans and horses^3,9^. Therefore, blocking proinflammatory T cell cytokines and enhancing anti-inflammatory T cell cytokines is an appealing target for uveitis treatment^4,12^.

One approach to immunomodulation, is to target the auto-pathogenic T-cells and inflammatory cytokines that orchestrate and exacerbate the disease process. The human leukocyte antigen-G (HLA-G) is an immunomodulatory and anti-inflammatory molecule that was first described to play a role in fetal protection against the maternal immune system^13–15^. HLA-G has multiple isoforms that exert multimodal strategies for specific or general immune suppression^13,15^. HLA-G inhibits the function of several types of immune cells, including directly inducing apoptosis of activated T cells and natural killer (NK) cells^13,14^. Additionally, HLA-G induces tolerogenic dendritic cells, and can promote the expansion of an immunosuppressive T cell subset by upregulating T regulatory cells (including CD4+ HLA-G+ T_reg_)^14,15^. HLA-G has been implicated in ocular and corneal immune privilege^16,17^ and in a previous study from our laboratories using AAV gene delivery, AAV-HLA-G1/5 effectively inhibited burn-induced neovascularization, immune cell infiltration, and fibrosis in the rabbit cornea^14^.

One potential approach to long-term drug delivery is the use of gene therapy, which is being developed as a specific targeted therapy for multiple genetic diseases and has been explored in detail for ocular diseases including glaucoma and inherited retinal diseases, including Luxturna, the first gene therapy biologic to be granted market approval^18–20^. Gene therapy based on adeno-associated virus (AAV) vectors is currently the most promising approach with a convincing safety profile in hundreds of treated patients. AAV transduces many different cell types and establishes long-term transgene production for years following a single injection^21,22^. AAV serotypes 1–12 have been described to have tropism for different ocular cell types and target tissues, presumably due to variations in capsid and host cell receptor affinities, nuclear trafficking, and/or uncoating^22–24^. Although there are numerous serotypes, AAV serotype 8 (AAV8) has been previously shown to effectively transduce the ocular uveal tissue^20–22,25,26^.

As recurrent NIU necessitates long-term immunosuppression to prevent vision loss, a single dose treatment that mediates long-term immunosuppression specifically in the target tissue without side effects is desired. Herein, we describe an endogenous human molecule capable of immune suppression at nearly all levels that is already implicated in ocular immune privilege (HLA-G) for the treatment of NIU using AAV gene therapy. To simulate the prevention of NIU, naïve Lewis rats received a single intravitreal injection of AAV particles harboring codon-optimized cDNAs encoding HLA-G1 and HLA-G5 isoforms one week prior to EAU induction. The results demonstrate that AAV-mediated expression of HLAG-1 and -5 gene therapy is an effective and novel gene therapy for the treatment of EAU.

## Results

### Gene delivery in EAU rats following intravitreal injection

**Figure 1:**
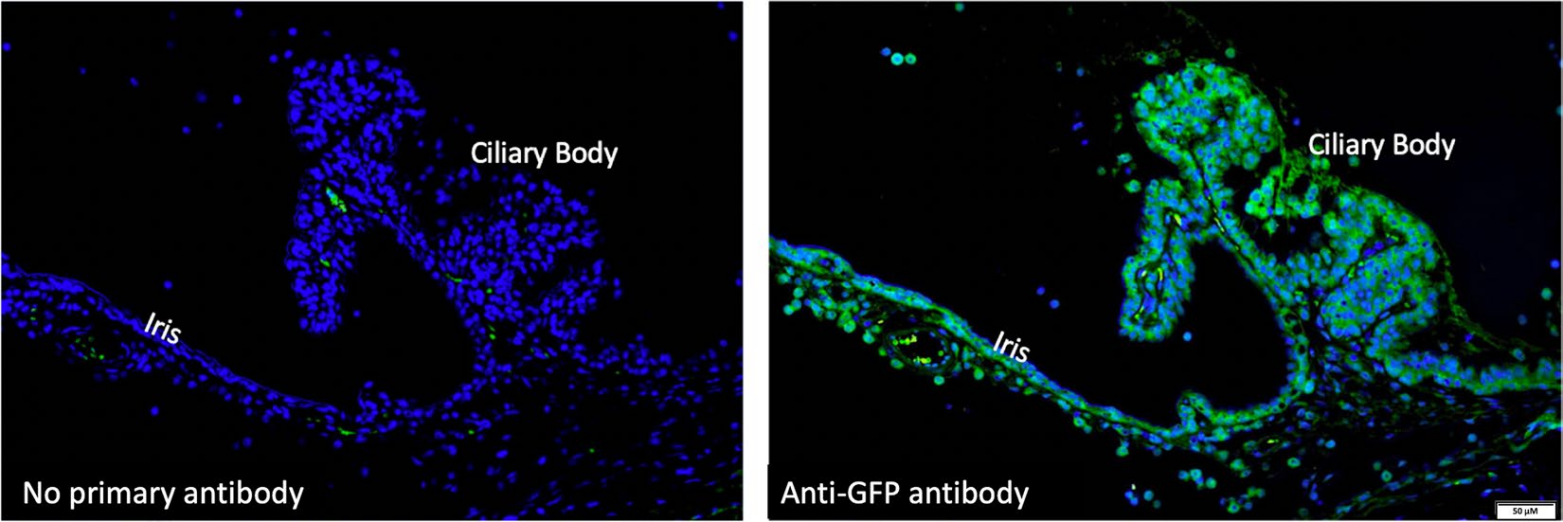
AAV8 gene delivery to EAU Iris and Ciliary Body. Representative histological images of GFP immunofluorescence following intravitreal injection of AAV8-GFP. Transgene expression (green) was examined by immunostaining. The left image is of the iris and ciliary body, without primary antibody; the right image is with anti-GFP antibody. To confirm gene delivery to inflamed uvea, rats were injected with either scAAV8-GFP (3 × 10^10^ viral genomes; 3 μL; n = 6) or balanced salt solution (3 μL; n = 3) and experimental autoimmune uveitis was induced in 3 of the AAV-GFP injected rats and 3 balanced salt solution rats one week following intraocular injections. Animals were euthanized 2 weeks following induction of EAU and tissues were collected for histopathology. The results demonstrate strong GFP staining in cells of inflamed ciliary body and iris following intravitreal injection of AAV8-GFP. The control section (left), without primary antibody, shows several focal areas of green fluorescence, assumed to be autofluorescence of red blood cells based on the anatomic location of the fluorescence.

In an initial preliminary study, rats were injected with either scAAV8-GFP (3 × 10^10^ viral genomes; 3 μL; n = 6) or balanced salt solution (3 μL; n = 3) intravitreally in the left eye and intracamerally in the right eye. Experimental autoimmune uveitis was induced in three of the AAV-GFP injected rats and three balanced salt solution rats one week following intraocular injections. Animals were euthanized two weeks following induction of EAU and tissues were collected for histopathology. This initial study in healthy and EAU rats demonstrated that intravitreal injection of AAV8-GFP resulted in greater transduction of the anterior uvea compared to the same vector/dose that was administered by intracameral injection. These results demonstrated strong GFP staining in cells of the inflamed ciliary body and iris in eyes injected intravitreally with AAV8-GFP (Fig. 1). Immunofluorescence of rats treated with intravitreal injections of AAV8-GFP without primary antibody and healthy eyes demonstrated minimal autofluorescence (Fig. 1). Transduction was also observed in the retina and corneal endothelium, but to a lesser extent than that seen in the iris and ciliary body.

### AAV-HLA-G1/5 gene therapy reduces inflammation in EAU rats

**Figure 2:**
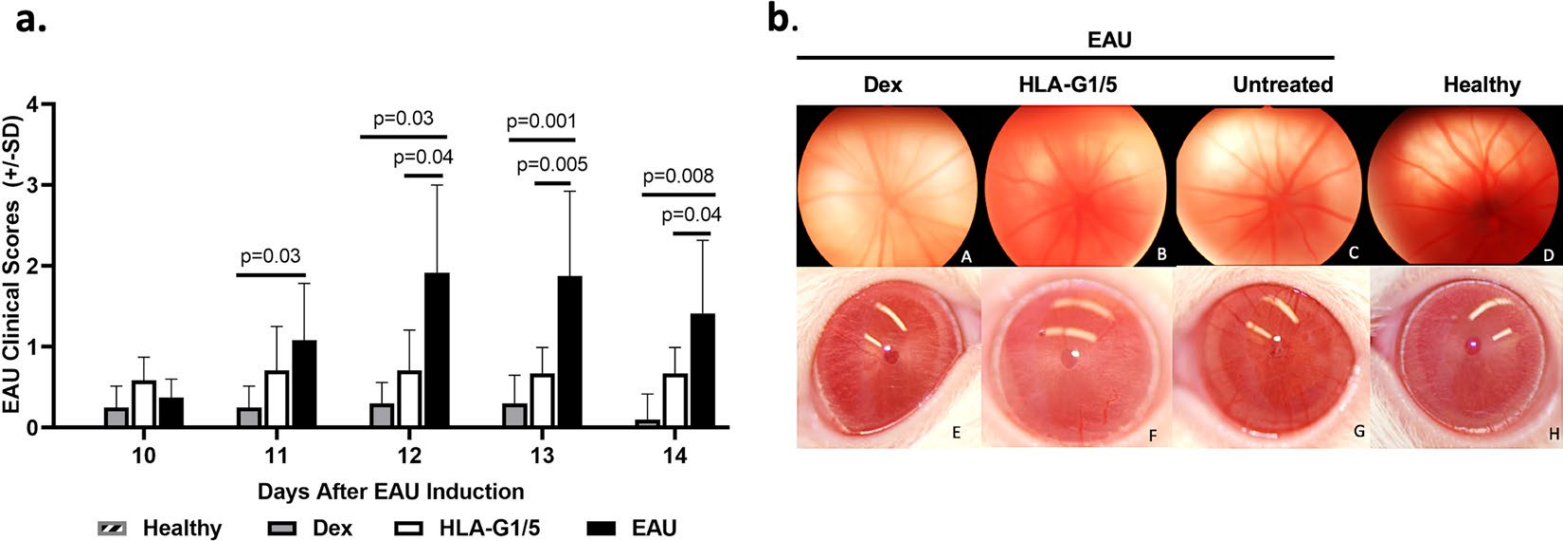
AAV8-HLA-G1/5 Improves EAU clinical scores. Rats were treated by intravitreal (IVT) injections with scAAV8-HLAG1/5 (n = 6 rats, 12 eyes) or topically treated with dexamethasone (Dex) (n = 5 rats, 10eyes); no treatment experimental autoimmune uveitis (EAU) rat (n = 6 rats, 12 eyes) and healthy rats (untreated, no EAU induction) (n = 3 rats, 6 eyes) were used as controls. One week after IVT injections, EAU was induced by immunization with IRBP and ocular inflammation was examined by slit lamp biomicroscopy. (**a**) Bar graph of EAU clinical scores revealed that EAU clinical scores peaked at days 12 and 13 after uveitis induction. Mean clinical scores were significantly less in eyes treated with a single intravitreal dose of scAAV8-HLA-G1/5 or topical dexamethasone (applied topically every 6 hours) compared to untreated EAU eyes on each day from 12 through 14 post-induction (P = 0.03 to 0.001); Pairwise Wilcoxon tests). There were no significant differences in mean clinical scores between eyes treated with scAAV8 HLA-G1/5 or dexamethasone at any day. Healthy eyes had inflammatory scores of 0 for each day of the study. (**b**) Retinal images (top row) taken on day 9 did not reveal any gross retinal damage in any treatment group; topical corticosteroid treatment (A), HLAG1/5 (B), EAU no treatment (C), healthy or control rat (D). Representative images taken on day 12 after EAU induction, demonstrate dilated iris blood vessels, hypopyon, fibrin, posterior synechia and severe miosis in EAU eyes with no treatment (G), whereas normal eyes were observed in eyes treated with topical corticosteroids (E) and HLAG (F) treated rats; Healthy eye (H).

In previous work from our laboratories, codon optimized cDNAs of human leukocyte antigen (HLA) G isoforms 1 and 5 inserted into AAV vectors prevented vascularization, fibrosis, and immune cell infiltration of the traumatized rabbit cornea^14^. As the anti-inflammatory effects of HLA-G in the eye are well established, HLA-G1/5’s function was evaluated for treatment of NIU using the EAU model in rats via AAV gene delivery. For these experiments self-complementary (sc) AAV8 vector preparations packaged with either HLA-G1 or HLA-G5 were used in combination at a ratio of 1:1 as described^14^. To simulate prevention of recurrent uveitis, healthy rats were treated with scAAV8-HLAG1/5 (total combined dose of 2.4 × 10^10^) vector genomes (vg) 7 days prior to EAU induction (N = 6 per group). EAU was then induced by immunization with IRBP as previously described^27^. Control groups included topical treatment with dexamethasone ophthalmic solution (30 μL of 0.1% solution) administered 4 times daily (following EAU induction) while the negative control cohort received no treatment. Ocular inflammation in EAU non-treated rats developed on day 10 after IRBP injection and peaked on days 12 and 13 (Fig. 2). Clinical EAU scores *(EAU Clinical scores: 0, normal; 0.5, dilated blood vessels in the iris; 1, abnormal pupil contraction; 2, hazy anterior chamber; 3, moderately opaque anterior chamber, but pupil still visible; 4, opaque anterior chamber and obscured pupil*^27^) revealed that intravitreal scAAV8-HLAG1/5 consistently resulted in attenuated ocular inflammation as compared to the untreated EAU rats (Fig. 2). Mean clinical scores were significantly less in eyes treated with intravitreal scAAV8-HLA-G1/5 or topical dexamethasone (every 6 hours) compared to untreated EAU eyes on each day from 12 through 14 post-induction (P = 0.03 to 0.001; Pairwise Wilcoxon tests, Fig. 2). There were no significant differences in mean clinical scores between eyes treated with scAAV8 HLA-G1/5 or dexamethasone at any day (Fig. 2). Healthy eyes had inflammatory scores of 0 for each day of the study.

The body weight of the rats at the end of the study that were treated with topical corticosteroids was a mean 156.0 g +/− 11.4 SD compared to the body weight of the scAAV8-HLA-G1/5 treated with a mean 221.67 g +/− 6.1 SD and untreated control rats with mean 220.8 g +/− 8.6 SD body weight. The average of 65.3 g less body weight in corticosteroid treated rats, plus the death of one corticosteroid treated rat on day 11, was considered to be the result of systemic absorption and adverse effects from the topical dexamethasone medication.

**Figure 3:**
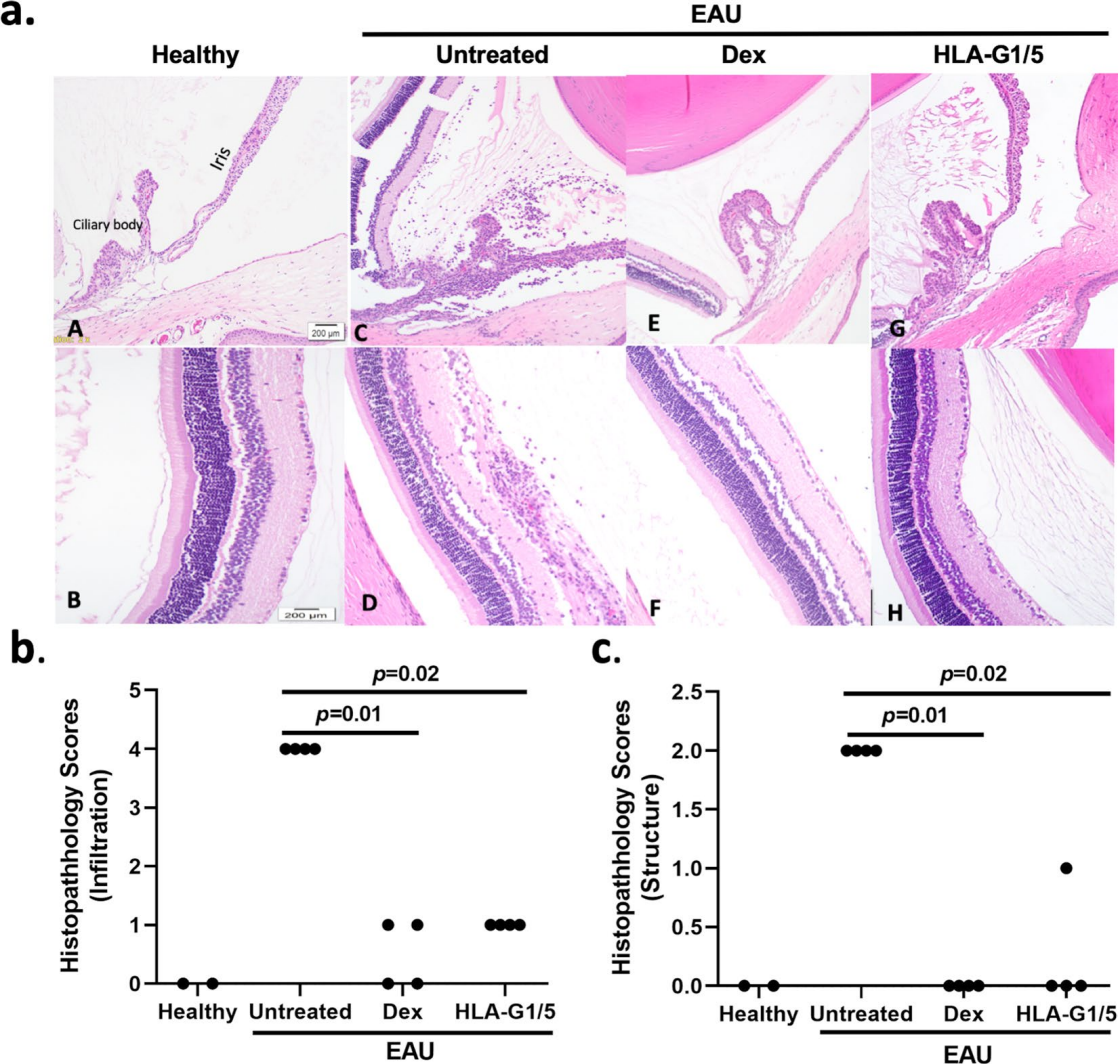
AAV8-HLA-G1/5 Improves EAU histology scores. (**a**) Representative images of ocular histology demonstrate iris thickening and severe inflammatory cell infiltration in the ciliary body, iris, anterior chamber and vitreous body, as well as moderate vasculitis formation in experimental autoimmune uveitis (EAU) untreated eyes (C,D). Mild infiltration of inflammatory cells was observed in the vitreous and retina in topical dexamethasone and HLA-G1/5 treated EAU eyes (E&F, G&H, respectively). Control healthy eyes (A&B). (hematoxylin & eosin staining, original magnification: 20x and 40x). The histological infiltrative scores (**b**) and structural scores (**c)** were significantly decreased in the scAAV8-HLA-G1/5 treated (n = 4 eyes) and dexamethasone (dex) treated EAU eyes (n = 4 eyes) as compared to the non-treated EAU eyes (n = 4 eyes) (p = 0.01, p = 0.02; Wilcoxon test). There was no significant difference between healthy eyes (n = 2 eyes) and either EAU treatment group. Each point is the average score of an individual eye of two blinded observers and mean scores of each treatment group are denoted by the horizontal bars.

At the conclusion of the clinical experiment (day 14 post-IRBP administration), ocular and non-ocular tissues were collected. Two rats from each treatment group (scAAV8 HLA-G1/5, dexamethasone, and EAU; total 4 eyes/group) and one healthy rat (total of 2 eyes) had both eyes fixed, sectioned, stained with H&E, and graded in a blinded manner by two experienced examiners using a well-established scoring system, which includes infiltrative and structure assessment and scores^4^. Histological examination in untreated EAU eyes showed a severe intraocular inflammation as evidenced by iris thickening, and severe inflammatory cell infiltration in the ciliary body, iris, aqueous humor and vitreous, as well as moderate vasculitis formation (Fig. 3). However, in the scAAV8-HLA-G 1/5 and topical dexamethasone treated eyes, only a few scattered inflammatory cells were observed (Fig. 3). The mean infiltrative and structural histological scores were significantly decreased in the scAAV8-HLA-G1/5 treated eyes (mean +/−SD 1.0 +/− 0.0 and 0.25 +/−0.5 [infiltrative and structural, respectively]) and in the topical corticosteroid treated EAU eyes (0.5 +/− 0.6; 0.0 +/− 0.0) as compared to the non-treated EAU eyes (4.0 +/− 0.0; 2.0 +/−0.0) (Kruskal-Wallis, Wilcoxon; p = 0.02; p = 0.02). The mean infiltrative and structural histologic scores of the healthy rat eyes were 0.0. There was no significant difference (p > 0.05) between healthy eyes and either treatment group (Fig. 3) (See Supplementary Table 1 for scoring scheme and individual histologic scores).

### HLA-G1/5 ocular expression and AAV8 biodistribution following intravitreal injection

**Figure 4:**
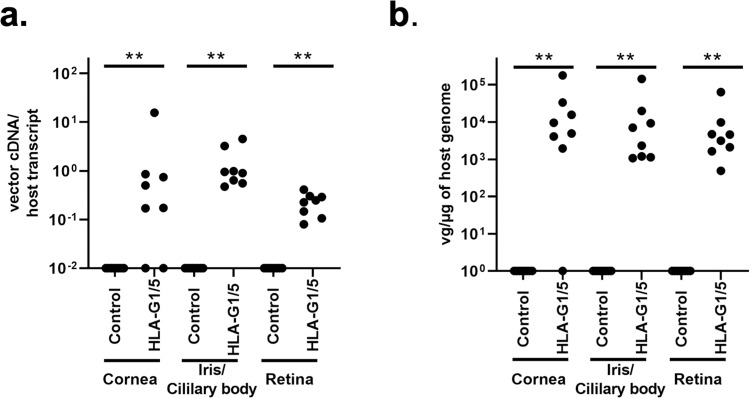
AAV8-HLAG1/5 expression and ocular distribution. AAV8-HLAG1/5 expression and ocular distribution. (**a**) HLA-G abundance examination by qRT-PCR in selected tissues are presented as vector cDNA /host transcript (GAPDH), n = 4 rats, 8 eyes, Mann-Whitney test, **p < 0.01. Results represent experiments done in triplicate with mean value expressed; (**b**) Vector genome copy number in distinct ocular tissues (cornea, iris/ciliary body, and retina) are shown as vector genome copy number/ug of host genome DNA, n = 4 rats, 8 eyes, Mann-Whitney test, **p < 0.01.

HLA-G1/5 expression in treated rats was confirmed by RT-QPCR using RNA recovered from the cornea, retina, and iris/ciliary body in 4 Rats (8 eyes) of each group. HLA-G transcript was detected in the ciliary body/iris and retina in all scAAV8-HLAG1/5 treated animals (Fig. 4a). HLA-G transcripts were also detected in RNA recovered in the cornea in the majority of scAAV8-HLA-G1/5 treated animals, however 25% of eyes (2 of 8 eyes) demonstrated no expression (Fig. 4a). scAAV8-HLA-G1/5 vectors demonstrated significant transduction in the cornea, ciliary body/iris, and retina compared to non-treated controls (p = <0.05, n = 8) (Fig. 4).

It is of importance for potential clinical applications to determine the biodistribution of persistent AAV vector genomes following scAAV8-HLA-G1/5 intravitreal injections and EAU induction. Therefore, DNA was extracted from the liver, kidney, brain, lymph nodes, and spleen, and QPCR was performed using primers specific for the transgenic cassette. Recombinant AAV genomes were not consistently detected in the non-ocular tissues examined, suggesting that AAV8-HLA-G1/5 biodistribution in peripheral organs of the EAU rat model was below the limit of detection (defined as 10 femtograms of viral genomes/0.2 ug of genomic DNA; Table S2). However, serum neutralizing antibodies to the AAV8 capsid were generated in all rats with the antibody titers at 50% neutralization listed in Table 1. Tissues from animals that did not receive AAV vectors were found negative for vector genomes as expected, and these animals did not present AAV8 capsid neutralizing antibodies. (Table 1, Supplementary Table 2 for the comprehensive dataset).

**Table 1:** Neutralizing antibody to AAV8.

Treatment	Serum Neutralizing antibody titer
AAV8-HLA-G1/5, EAU	256
AAV8-HLA-G1/5. EAU	4
AAV8-HLA-G1/5, EAU	512
AAV8-HLA-G1/5, EAU	256
AAV8-HLA-G1/5, EAU	128
AAV8-HLA-G1/5, EAU	256
EAU, no treatment	ND
EAU, no treatment	ND
EAU, no treatment	ND
EAU, no treatment	ND
EAU, no treatment	ND
EAU, no treatment	ND
No EAU, No treatment	ND
No EAU, No treatment	ND

The neutralizing antibody titer as 50% of transduction inhibition was listed for each Rat.

ND: Not detectable.

## Discussion

Uveitis remains a significant cause of vision loss in humans with approximately 30,000 new cases diagnosed each year in the Unites States alone^28,29^. Non-infectious uveitis (NIH), which is the most common type of uveitis in the United States (prevalence of 121 cases in 100,000 adults, and 129 cases in 100,000 juveniles), is recurrent in its nature and can result in secondary complications such as cataracts, corneal opacities, glaucoma, and blindness^18,30^. Because NIU is a multifactorial disease, systemic corticosteroids and other immunosuppressive therapeutics have been employed clinically as the current mainstay treatment option^2,31^. Such treatments however, are non-specific and have serious side effects which limits them to short term use, and therefore are ineffective as a preventative for recurrent bouts of inflammation^18^. Gene therapy is a promising treatment for NIU. Following a single, localized treatment, AAV-HLA-G would reduce or eliminate the need for long-term corticosteroids while moderating ocular inflammation and preventing secondary tissue destruction in NIU^14,22^. AAV vectors, in particular, offer a potentially powerful approach for the treatment of recurrent inflammation, by introducing long term sustained intraocular delivery of therapeutic proteins like HLA-G1/5 at the ciliary body and iris, the targeted sites of the blood ocular barrier breakdown in uveitis^1,22^.

In the present study, we observed that intravitreal injection of AAV8-HLA-G1/5 resulted in a significant decrease in ocular inflammation in the EAU rat model. These results were correlated to robust expression of HLA-G1/5 cDNA at the cornea, ciliary body/iris and retina (see Fig. 4). HLA-G’s induction of immune tolerance is exerted directly via HLA-G1’s inhibitory interactions with nearly all immune cells and indirectly by inhibiting vascularization via the soluble HLA-G5 isoform, the combination of which is hypothesized to prevent self-antigen recognition and immune response^13^. Previous studies in autoimmunity have found that HLA-G expression reduced the incidence of acute and chronic heart transplant rejection in humans^32^ and that a single injection of endogenously produced HLA-G could be used as a tolerogenic molecule in skin transplants in animal models^13^. These studies demonstrated that HLA-G inhibits T cell functions and may attenuate clinical rejection by influencing a shift in cytokine expression profiles toward Th2 and production of TNFA, IFN-γ, and IL-10, endogenous immunomodulatory cytokines with potent immunosuppressive functions^32,33^. Additionally, the combination of HLA-G1 and HLA-G5 was demonstrated to provide long-term immunosuppressive in corneal inflammation and vascularization of rabbits^14^. In agreement with these studies, our data of scAAV8-HLA-G1/5 treated EAU rats had significantly reduced clinical and histological inflammatory scores following a single intravitreal injection. To the authors’ knowledge this is the first report to demonstrate the use of HLA-G1/5 as an intraocular immunosuppressive therapeutic.

This study also evaluated topical corticosteroids, a commonly-used therapeutic in the clinic, as a treatment control to compare anti-inflammatory efficacy to intravitreal scAAV8-HLA-G1/5 in EAU rats. The data demonstrated that both treatment groups were able to significantly ameliorate signs of EAU, including a significant difference of clinical and histologic inflammatory scores between EAU with no treatment and both treatment groups (topical corticosteroids and scAAV8-HLA-G1/5) as well as a significant difference between clinical scores between healthy rats with no treatment and both treatment groups. Although clinical and histological inflammatory scores of rats treated with topical corticosteroids were significantly lower than the scAAV8-HLA-G1/5 treated eyes, it is of note that the topical ocular corticosteroids were being dosed four times a day, while the scAAV8-HLA-G1/5 was a single intravitreal injection 1 week prior to the induction of EAU. Additionally, rats treated with topical corticosteroids demonstrated adverse systemic effects, including weight loss (mean of approximately 65 g less than other groups) and death, thought to be associated with systemic absorption of the corticosteroid and possibly stress associated with repeated handling required for dosing. Progressive weight loss of >60 g has also been documented previously in normal rats after 1 week of topical ocular 0.1% dexamethasone which continued to over 100 g weight loss after 4 weeks^34^. This weight loss was accompanied by increased cholesterol and liver enzymes (alanine transaminase [ALT]), therefore systemic absorption of the dexamethasone was considered causative^34^. Though systemic absorption of topical ocular drugs in larger animals and humans may have less of an impact than in rodents, it is an important reminder of the side effects of topical corticosteroid treatment. The dose, volume and environment (healthy vs inflamed eye) in which the scAAV8-HLA-G1/5 is injected intravitreally will need to be explored in future studies to optimize its therapeutic effects.

AAV8 intravitreal injections have been well characterized in animal models and also have been used in humans for the treatment of X-linked retinoschisis^35,36^. The data herein demonstrate in rats that intravitreal injections of AAV8 prior to the induction of EAU, resulted in significant transduction of the iris/ciliary body with signal also detected in other ocular tissue by qPCR (Fig. 4). This finding is consistent with a previous report using very high dose AAV8 intravitreal injections in WT mice^35^. However, results from the sensitive HLA-G expression analysis using RT-PCR demonstrated near equal amounts of cDNA produced among the uvea, cornea, and retina following intravitreal injection of scAAV8-HLA-G1/5, perhaps highlighting the less sensitive nature of immunofluorescence compared to RT-PCR. Given this result, along with concerns regarding the overexpression of HLA-G in some neoplasms, future experimentation is intended to compare the therapeutic effects observed herein to new constructs using ubiquitous and ciliary body-restricted promoters. However, it is noteworthy that HLA-G expression is naturally found in the human retina and cornea^33^, decreasing concerns of adverse effects from transducing ocular tissues other than the uveal tract.

Despite the promising results described above, our study has some limitations. First, although the safety of AAV-mediated gene therapy of ocular diseases has been established in clinical trials and we did not observe any adverse effects in our study, it is unknown whether long-term ocular HLA-G1/5 expression would result in ocular or systemic adverse effects or promote development of neoplasia. Although it is possible that any immunosuppression may increase susceptibility of ocular infections, because HLA-G is a naturally a component of ocular immune privilege, it is likely that AAV-HLA-G therapy helps to restore the natural ocular immune privilege, not overtly increase risk of infections, at a level less than the use of standard of care topical corticosteroids. Hence, a long-term safety study of local HLA-G is needed, perhaps using a uveal-restricted promoter, prior to clinical use.

Secondly, the therapeutic control group of rats were treated with topical ocular corticosteroids, instead of systemic corticosteroids. In many patients, systemic corticosteroids are avoided due to non-specific immune suppression and other dangerous side effects. Although topical ocular corticosteroid therapy is commonly used to treat NIU, it isn’t used as frequently as systemic corticosteroids because frequent topical ocular administration is limited by poor patient compliance. Therefore, it would have been interesting to compare systemic corticosteroid use to intravitreal scAAV8-HLA-G1/5 in rat EAU model.

In summary, we have demonstrated that intravitreal delivery of a single dose of scAAV8-HLA-G1/5 suppressed ocular inflammation in EAU rats. ﻿ In contrast to systemic and topical immune suppression, localized gene delivery of HLA-G may significantly reduce the off-target risks and establish a long-term anti-inflammatory effect that would be an effective, novel therapeutic strategy for refractory and recurrent uveitis as well as other ocular autoimmune inflammatory diseases in both human and veterinary patients.

## Methods

### Vector production and purification

The codon optimized HLA-G1 and HLA-G5 cDNAs were described previously^14^. Self-complementary (sc) AAV8-Jet-HLA-G1, scAAV8-Jet-HLA-G5, and AAV8-CMV-GFP vector preparations were produced by the UNC Vector Core and characterized prior to use, as previously described^14^. The Jet promotor, which is a ubiquitous promoter, was used in these constructs, as described in detail previously.

### Animals

These studies using adult female Lewis rats were reviewed and approved by the *North Carolina State University Institutional Animal Care and Use Committee* (IACUC) and performed according to the Association for Research in Vision and Ophthalmology Statement for the Use of Animals in Ophthalmic and Visual Research. Animals were housed under 12/12 hour light/dark cycle in the NC State Laboratory Animal Resources facility.

In an initial preliminary study, rats were randomly divided into 3 groups: the first (n = 3) and second group (n = 3) were injected with scAAV8-GFP at a dose of 3 × 10^10^ viral genomes (vg) intravitreally in the left eye and intracamerally in the right eye, while the third group (n = 3) was injected with balanced salt solution (3 μL final volume) intravitreally in the left eye and intracamerally in the right eye. Experimental autoimmune uveitis was induced in groups two and three one week following intraocular injections. Animals were euthanized two weeks following induction of EAU and tissues were harvested for histopathology, described below.

In the second part of this study, rats were randomly divided into 4 groups: the first group (n = 6) had both eyes injected intravitreally with scAAV8 vectors encoding a combination of HLA-G1 + HLA-G5 at a 1:1 ratio, a total dose of approximately 2.4 × 10^10^ viral genomes (vg) with a total volume 3 μL). The second group of rats (n = 6) were treated with 30 μL topical Neomycin, Polymyxin, Dexamethasone (0.1%) ophthalmic solution (Bausch and Lomb) 4x a day. The third group (n = 6) and fourth group (n = 3) of rats received no ocular treatments. Experimental autoimmune uveitis was induced in Groups 1–3 one week following intraocular injections. The fourth group of rats were used as a healthy control. Rat serum for neutralizing antibody analysis was obtained from the lateral tail vein before intraocular injection and then obtained via intracardiac blood draw immediately after euthanasia. Serum collected was stored at −80 °C. Daily slit lamp examinations assessed ocular abnormalities induced by the intraocular injections and following induction of EAU. Animals were sacrificed two weeks following induction of EAU and tissues were harvested for further analyses, as described below. One rat from the topical ocular dexamethasone-treated rats died on day 11; therefore, data from this rat was not used for analysis.

### Intravitreal administration of scAAV8-HLA-G1/5

Prior to vector delivery, rats were anesthetized with 2–3% Isoflurane (Henry Schein) in oxygen to effect. Topical anesthetic, proparacaine HCL 0.1% (Bausch and Lomb) was applied to the eyes prior to intraocular injection. Animals were placed in lateral recumbency (left eye injected first followed by the right eye). Each eye was cleaned with dilute Betadine solution. Intraocular injections were performed under an operating microscope using a polyethylene tubing (I.D. 0.38 mm, O.D 1.09 mm) connected to a Hamilton syringe (Hamilton) and a 34 G stainless steel needle. Three microliters viral suspension (2.4 × 10^10^ vg) mixed with 0.01% fluorescein sodium salt (Sigma) was administered intravitreally in both eyes, with the needle placement 1–2 mm posterior to the temporal limbus. After injections were completed, topical antibiotic solution, Moxifloxacin 0.5% (Apotex Corp.) and topical ocular lubrication was applied to the ocular surface to prevent infection and desiccation and rats were kept on a heating pad until fully awake.

### EAU induction and clinical evaluation of EAU

All EAU rats were induced seven days post intraocular injections of scAAV8 to permit peak transgene expression and inflammation at approximately the same time. All rats except the control healthy rat group (group 4), were immunized subcutaneously at the base of the tail (100 µg) and both thighs (50 µg) with a 1:1 volume of human interphotoreceptor retinoid binding protein (IRBP) and Complete Freund’s Adjuvant (CFA).

Blinded clinical assessment by slit lamp biomicroscopy examination was performed daily by the same examiner. Each eye was graded, according to a previously described scoring system: 0, normal; 0.5, dilated blood vessels in the iris; 1, abnormal pupil contraction; 2, hazy anterior chamber; 3, moderately opaque anterior chamber, but pupil still visible; 4, opaque anterior chamber and obscured pupil^27^. Clinical scores for each individual eye were given one value per eye per day.

### Whole body AAV biodistribution

To investigate the biodistribution of AAV vectors delivered via intravitreal injections in an EAU model, rat tissue was harvested three weeks following intravitreal injections following strict tissue collection and instrument cleaning procedures to minimize the potential for cross-contamination. Healthy rats were dissected first followed by rats treated with topical dexamethasone, then rats with intraocular injections. Different sets of instruments were used for intraocular and extraocular tissue, and for different groups of rats. Instruments were cleaned with alcohol, 5% Sodium dodecyl sulfate detergent (5% SDS), and sterile saline between each sample taken. A board certified ophthalmologist dissected all intraocular tissues under an operating microscope. Upon collection, tissues were frozen on dry ice and then stored at −80 °C. DNA from the liver, kidney, spleen, lymph nodes, and brain were isolated using a DNeasy Blood and Tissue Kit (Qiagen, Valencia, CA). Total DNA concentration was quantified via NanoDrop and subsequently diluted with nuclease-free water to a working concentration of 10 ng/uL. Vector genome copy number was analyzed by quantitative PCR (QPCR) based on Universal Probe Library (UPL) technology as previously described^14,22^. Briefly, the vector-specific HLA-G target was detected using the UPL probe #26 and the following primers: forward primer: tcttggaccgcagcagata; reverse primer: gtttgctgcctcgcactt. QPCR reactions were prepared with the LightCycler 480 2X Probes Master Mix (Roche, Mannheim, Germany), 0.2 µM Universal Probe Library Probe 26 (Roche, Mannheim, Germany), 0.5 µM forward primer, 0.5 µM reverse primer, and a total of 200 ng of template genomic DNA diluted to a final reaction volume of 50 uL with nuclease-free water. Serial dilutions of HLA-G5 plasmid DNA ranging from 0.0001–1000 pg were used to generate a standard curve. Reactions were performed at an annealing temperature of 56 °C on a LightCycler 480 quantitative PCR instrument (Roche, Mannheim, Germany). Two independent experiments with were performed for each tissue in triplicate and the average Cp value was used to calculate the quantity of PCR product (in femtograms) present in each sample from the standard curve. The limit of detection (10 femtograms of viral genome/0.2 ug of genomic DNA) was determined by selecting the lowest amount of plasmid DNA that could be reproducibly detected in the linear portion of the standard curve from four independent experiments. Samples that were outside the linear range of the standard curve were considered to be below the limit of detection (<10 fg = Threshold (Cp) values were below the limit of detection, N.D. = not detected). Viral genome copy number was then determined using the following formula^37^. All results (including replicates) are provided in Supplementary Table 2.

$${\rm{Copy}}\,{\rm{number}}=\frac{fg\ast 6.022\ast {10}^{23}}{650\ast size\,of\,plasmid(bp)\ast {10}^{15}}$$

### Quantification of vector biodistribution and transgene expression in ocular tissues

Following enucleation, 8 eyes (4 rats) from each group were dissected immediately to separate the cornea, iris/ciliary body and retina, then frozen and stored at −80 °C until processing. Two rats (Four eyes) from groups 1–3 and 1 rat (2 eyes) from group 4 were fixed in 4% buffered paraformaldehyde for histopathology. DNA/RNA from the cornea, iris/ciliary body and retina were extracted with the AllPrep DNA/RNA Mini Kit according to the kit protocol (Qiagen, Valencia, CA). All experiments were performed in triplicate. Vector genome copy number and transgene expression level were quantitatively analyzed by qPCR and qRT-PCR, respectively based on the UPL technology described above^14,22^. The HLA-G viral genome copies and cDNA level were standardized against an amplicon from a single copy rat gene, GAPDH, amplified from genomic DNA or cDNA using the same primer/probe sets as the AAV whole body biodistribution study described in the previous section. Vector genome biodistribution data are reported as the number of vector genome copies per µg of gDNA, and the transgene expression level is reported as vector cDNA (HLA-G1/5)/host transcript. Data was plotted using GraphPad Prism v8.2.1 software.

### Histopathological evaluation of EAU and scoring

Animals were euthanized during peak disease activity (day 14 after induction of EAU), and the eyes were enucleated. Two rats (Four eyes) from groups 1–3 and 1 rat (2 eyes) from group 4 were fixed in 4% buffered paraformaldehyde overnight at 4 °C and then transferred to 70% ethanol before embedding in paraffin. Eyes were sectioned 5 µm through the optic nerve horizontal plane, and stained with hematoxylin and eosin. Blinded infiltrative and structural grades of each eye were scored as previously described^4^. The globes were scored by two blinded observers separately and averaged to determine infiltrative and structural scores for each rat. Data are presented as mean +/− standard deviation (SD) (Fig. 3) (See Supplementary Table 1 for scoring scheme and individual scores).

### Immunofluorescence for transgene expression

Immunofluorescence was performed following a previously described method^14^. In short, sections were deparaffinized by incubating the slides two times in xylene for 10 min each, followed by immersing the slides sequentially in two rounds of 100% (3 min each), 95% (1 min), and 80% (1 min) ethanol solutions, and finally in water for 5 min. Antigen retrieval procedure was performed by heating the slides to 95 °C in citrate-based (pH 6.0) antigen unmasking buffer (Vector Laboratories) before staining. Non-specific staining was blocked by using PBS containing 10% normal goat serum, 0.025% Triton X-100, and 1% BSA before overnight incubation with primary antibody. The GFP primary antibody (1:500 dilution, AVES Labs, GFP-1010) and goat anti-chicken secondary antibody (Alexa Fluor 488, 1:1000, Abcam, A11039) were used to locate GFP expression. After the staining, slides were mounted and counter-stained with ProLong Diamond Antifade Mountant with DAPI ﻿(p36971, Invitrogen) and observed with an Olympus IX83 Fluorescence Microscope (Olympus, Tokyo, Japan) or Zeiss LSM 780 inverted confocal microscope.

### AAV neutralizing antibody assay

To determine if intravitreal injection of AAV vectors results in an antibody response to the injected capsid, neutralizing antibody assays were performed on HEK 293 cells using a previously reported method^14^. In short, cells were seeded in a 48-well plate at 25,000 cells/well in duplicate on the day prior to vector transduction. The next day, the pre- and post-injection serum was used 1:1 and then serially diluted 1:2 to 1:512 in DPBS to a final volume of 13 ul and incubated with AAV8 CMV-Firefly Luciferase titered to 8 × 10^8^ total viral genomes per replicate in 13 ul DPBS for 2 hours at 4 °C. Serum/vector mixture was then added to cells and a luciferase assay was performed 48 hours post-transduction using Promega Luciferase Assay System (Bright-Glo; Promega, Madison, WI) using a Perkin Elmer Victor 3 1420 Multi-label Counter Luminometer. Results were plotted to find the point at which the serum dilution suppressed transduction to less than 50% of pre-injection serum levels.

### Statistics

Group numbers of rats were estimated using power statistics described by Lenth^38^. Comparisons of clinical and histologic scores from the experiments in this study were analyzed initially using the non-parametric Kruskal-Wallis test to evaluate clinical and histologic scores, i.e., to determine if at least one sample dominated. If there was a significant difference, then pairwise Wilcoxon (Mann-Whitney) tests were performed to evaluate for group differences using JMP version 14.0 (SAS Institute Cary, NC). QPCR data generated from the experiments in this study were analyzed with GraphPad Prism, version 8.0, using the Mann-Whitney test. Significance was set at p < 0.05 for all comparisons in this study^39^.

## Supplementary information

Supplementary Information
