# Supplementary material for: AAV-mediated expression of HLA-G1/5 reduces severity of experimental autoimmune uveitis
Source: Sci Rep. 2019 Dec 27;9:19864. doi: 10.1038/s41598-019-56462-3 (PMC6934797; doi:10.1038/s41598-019-56462-3)
Supplement: Supplementary file 1 — Supplementary Information [file 41598_2019_56462_MOESM1_ESM.pdf]

AAV-mediated expression of HLA-G1/5 reduces severity of experimental autoimmune uveitis

Elizabeth Crabtree,[1] Liujiang Song,[2,3,4] Telmo Llanga,[3,4] Jacquelyn J. Bower,[3,4,5] Megan Cullen,[1] Jacklyn H. Salmon,[1] Matthew L. Hirsch,[3,4] Brian C. Gilger[1]

1. College of Veterinary Medicine, North Carolina State University, Raleigh, NC USA,

2. Department of Pediatrics, Hunan Normal University Medical College, Changsha, Hunan, China,

3. Ophthalmology, University of North Carolina, Chapel Hill, NC, USA.

4. Gene Therapy Center, University of North Carolina, Chapel Hill, NC, USA.

5. Lineberger Comprehensive Cancer Center, University of North Carolina, Chapel Hill, NC, USA

Supplementary tables

Supplementary Table 1 – Histologic Scores

	Cellular Infiltration (score range)[a]									Structural / morphologic changes[a] (score range)			
**Rat / treatment group**	Iris (0-4)	Anterior chamber (0-4)	Cornea (0-2)	Ciliary body (0-4)	Vitreous (0-5)	Vasculitis (0-5)	Rod outer segments (0-5)	Choroid (0-4)	Granulomas (0-3)	Rod outer segments (0-4)	Neuronal layers (0-5)	Retinal morphology (0-4)	SRNVM (0-2)
1 HLA-G	0	0	0	0	1	0	0	0	0	0	0	0	0
2 HLA-G	0.5	1	0	0	1	0.5	0	0	0	0	0	0	0
3 HLA-G	0.5	0	0	.5	1	0	0	0	0	0	0	0	0
4 HLA-G	1.5	1	0	1	1.5	0.5	0.5	0.5	0	1	0	0	0
1 Dex	0	0	0	0	0	0	0	0	0	0	0	0	0
2 Dex	0	0	0	0	1	0	0	0	0	0	0	0	0
3 Dex	0	0	0	0	1	0	0	0	0	0	0	0	0
4 Dex	0	0	0	0	0	0	0	0	0	0	0	0	0
1 EAU	4	4	1.5	4	5	5	3	1	2	3	0	0	0
2 EAU	3	4	1	3	4.5	4	2	2	2	2	1	1	0
3 EAU	4	4	1	4	5	4.5	3	1	2	3	1	1	0
4 EAU	3	3.5	1	3.5	4	4	2	0	2	2	0	0	0
1 Healthy	0	0	0	0	0	0	0	0	0	0	0	0	0
1 Healthy	0	0	0	0	0	0	0	0	0	0	0	0	0

[a]Average score of two blinded reviewers

Supplementary Table 1 – Histologic Scores

Histology Scores

	Cumulative scores		Grades[b]		Group means +/- SD	
**Group**	**Infiltrative**	**Structural**	**Infiltrative**	**Structural**	**Infiltrative**	**Structural**
**1 HLA-G**	1	0	1	0	1.0 +/- 0	0.25 +/- 0.5
**2 HLA-G**	3	0	1	0		
**3 HLA-G**	2	0	1	0		
**4 HLA-G**	6	1	1	1		
**1 Dex**	0	0	0	0	0.5 +/- 0.6	0.0 +/- 0.0
**2 Dex**	1	0	1	0		
**3 Dex**	1	0	1	0		
**4 Dex**	0	0	0	0		
**1 EAU**	29.5	3	4	2	4.0 +/- 0.0	2.0 +/- 0.0
**2 EAU**	25.5	4	4	2		
**3 EAU**	28.5	5	4	2		
**4 EAU**	23	2	4	2		
**1 Healthy**	0	0	0	0	0.0 +/- 0.0	0.0 +/- 0.0
**2 Healthy**	0	0	0	0		

[b]Grades: Infiltrative: 0: none; 1: <10; 2: 10-15; 3: 15-20; 4: 20-30; 5: 30-35; 6: >35
Structural: 0: none; 1: <2; 2: 2-6; 3: 6-10; 4: 10-14; 5: 14

Supplementary Table 2: AAV Biodistribution in Select Rat Tissues

**AAV-HLA-G Treated Rats**	**Rat #16**			**Rat #17**			**Rat #18**			**Rat #19**			**Rat #20**			**Rat #21**		
**Replicate #**	**1**	**2**	**3**	**1**	**2**	**3**	**1**	**2**	**3**	**1**	**2**	**3**	**1**	**2**	**3**	**1**	**2**	**3**
Brain Expt #1	N.D	N.D	N.D	N.D	N.D	N.D	N.D	N.D	N.D	N.D	N.D	N.D	N.D	N.D	N.D	N.D	N.D	N.D
Brain Expt #2	<10 fg	<10 fg	<10 fg	<10 fg	<10 fg	<10 fg	N.D.	N.D.	N.D.	N.D.	N.D.	N.D.	N.D.	N.D.	N.D.	N.D.	N.D.	<10 fg
Kidney Expt #1	N.D	N.D	N.D	N.D	N.D	N.D	N.D	N.D	N.D	N.D	N.D	N.D	N.D	N.D	N.D	N.D	<10 fg	468
Kidney Expt #2	N.D.	N.D.	N.D.	N.D.	N.D.	N.D.	N.D.	208	N.D.	N.D.	N.D.	N.D.	N.D.	N.D.	N.D.	N.D.	N.D.	N.D.
Liver Expt #1	N.D	N.D	N.D	N.D	N.D	N.D	N.D	N.D	N.D	N.D	N.D	N.D	N.D	N.D	N.D	N.D	N.D	N.D
Liver Expt #2	N.D.	N.D.	N.D.	<10 fg	<10 fg	<10 fg	N.D.	N.D.	N.D.	<10 fg	<10 fg	<10 fg	N.D.	<10 fg	N.D.	N.D.	<10 fg	<10 fg
Spleen Expt #1	1120	N.D	N.D	N.D	N.D	N.D	N.D	N.D	N.D	N.D	N.D	N.D	N.D	N.D	N.D	N.D	N.D	N.D
Spleen Expt #2	<10 fg	<10 fg	<10 fg	N.D.	N.D.	N.D.	N.D.	N.D.	N.D.	N.D.	N.D.	N.D.	N.D.	N.D.	N.D.	N.D.	N.D.	N.D.
Lymph Node Expt #1	N.D	N.D	N.D	N.D	N.D	N.D	N.D	N.D	N.D	N.D	N.D	N.D	N.D	N.D	N.D	N.D	N.D	N.D
Lymph Node Expt #2	<10 fg	<10 fg	<10 fg	<10 fg	<10 fg	<10 fg	<10 fg	<10 fg	<10 fg	<10 fg	<10 fg	<10 fg	<10 fg	<10 fg	<10 fg	<10 fg	<10 fg	<10 fg

Supplementary Table 2: AAV Biodistribution in Select Rat Tissues

**Untreated Rats (Control)**	**Rat #40**			**Rat #41**			**Rat #42**		
**eplicate #**	**1**	**2**	**3**	**1**	**2**	**3**	**1**	**2**	**3**
Brain Expt #1	N.D	N.D	N.D	N.D	N.D	N.D	N.D	N.D	N.D
Brain Expt #2	N.D	N.D	<10 fg	N.D	<10 fg	<10 fg	N.D	N.D	N.D
Kidney Expt #1	N.D	N.D	N.D	N.D	N.D	N.D	N.D	N.D	N.D
Kidney Expt #2	N.D	N.D	N.D	N.D	N.D	N.D	N.D	N.D	N.D
Liver Expt #1	N.D	N.D	N.D	N.D	N.D	N.D	N.D	N.D	N.D
Liver Expt #2	N.D	N.D	<10 fg	N.D	<10 fg	<10 fg	N.D	N.D	N.D
Spleen Expt #1	N.D	N.D	N.D	N.D	N.D	N.D	N.D	N.D	N.D
Spleen Expt #2	N.D	N.D	N.D	N.D	N.D	N.D	N.D	N.D	N.D
Lymph Node Expt #1	N.D	N.D	N.D	N.D	N.D	N.D	N.D	N.D	N.D
Lymph Node Expt #2	<10 fg	<10 fg	<10 fg	<10 fg	<10 fg	<10 fg	<10 fg	<10 fg	<10 fg

Supplementary Table 2: AAV biodistribution results from selected rat tissues. All values are expressed as AAV viral genome copy number/ug of total host genomic DNA input (200 ng, as measured by Nanodrop). Three samples were detected as positive: Rat #21 Experiment #1 Replicate 3, Rat #18 Experiment #2 Kidney Replicate 2, and Rat #16 Experiment #1 Spleen Replicate 1. Due to the irreproducibility of these replicates (only 1/6 were positive in two independent experiments), these samples are likely the result of outside contamination.

<10 fg: Threshold (Cp) values were below the limit of detection of this assay, and considered as false positives.

Yellow highlighting indicates that the detector call was uncertain.

N.D. = Not Detectable, Cp >40.
